# An efficient approach to BAC based assembly of complex genomes

**DOI:** 10.1186/s13007-016-0107-9

**Published:** 2016-01-20

**Authors:** Paul Visendi, Paul J. Berkman, Satomi Hayashi, Agnieszka A. Golicz, Philipp E. Bayer, Pradeep Ruperao, Bhavna Hurgobin, Juan Montenegro, Chon-Kit Kenneth Chan, Helena Staňková, Jacqueline Batley, Hana Šimková, Jaroslav Doležel, David Edwards

**Affiliations:** School of Agriculture and Food Science, University of Queensland, Brisbane, QLD 4072 Australia; Centre for Biotechnology and Bioinformatics, College of Biological and Physical Sciences, University of Nairobi, P. O. Box 30197, Nairobi, 00100 Kenya; CSIRO Plant Industry, Brisbane, QLD 4072 Australia; School of Plant Biology, University of Western Australia, Perth, WA 6009 Australia; Institute of Experimental Botany, Centre of the Region Haná for Biotechnological and Agricultural Research, Šlechtitelů 31, 78371 Olomouc, Czech Republic

**Keywords:** Next-generation sequencing, SASSY, BAC, Assembly, 7DS, *Triticum aestivum*, *Saccharum spp*

## Abstract

**Background:**

There has been an exponential growth in the number of genome sequencing projects since the introduction of next generation DNA sequencing technologies. Genome projects have increasingly involved assembly of whole genome data which produces inferior assemblies compared to traditional Sanger sequencing of genomic fragments cloned into bacterial artificial chromosomes (BACs). While whole genome shotgun sequencing using next generation sequencing (NGS) is relatively fast and inexpensive, this method is extremely challenging for highly complex genomes, where polyploidy or high repeat content confounds accurate assembly, or where a highly accurate ‘gold’ reference is required. Several attempts have been made to improve genome sequencing approaches by incorporating NGS methods, to variable success.

**Results:**

We present the application of a novel BAC sequencing approach which combines indexed pools of BACs, Illumina paired read sequencing, a sequence assembler specifically designed for complex BAC assembly, and a custom bioinformatics pipeline. We demonstrate this method by sequencing and assembling BAC cloned fragments from bread wheat and sugarcane genomes.

**Conclusions:**

We demonstrate that our assembly approach is accurate, robust, cost effective and scalable, with applications for complete genome sequencing in large and complex genomes.

**Electronic supplementary material:**

The online version of this article (doi:10.1186/s13007-016-0107-9) contains supplementary material, which is available to authorized users.

## Background

Genome sequencing is revolutionising our understanding of biology, and the field is developing rapidly due to advances in DNA sequencing technologies. However, as a greater number of genomes are sequenced, there has been a general decline in the quality of published whole genome shotgun assemblies due to gaps and miss-assemblies [1–3]. Many biological questions can be answered without the need of a gold standard pseudo molecule reference assembly. For example, the analysis of gene content, the discovery and application of molecular genetic markers and evolutionary studies can be undertaken with draft whole genome shotgun assemblies which are relatively quick and inexpensive to produce. However, these draft assemblies have limitations, particularly in complex and polyploid genomes where it is difficult to resolve paralogues or homoeologues. Finished pseudo molecules are also required for the detailed study of genome rearrangements. The production of at least one high quality reference assembly should be a goal for all major crop plants.

Despite recent advances, the production of reference genomes remains hampered by factors such as a high repeat content, gene and genome duplication [4, 5]. Incorporating repeat spanning mate pair (MP) data and newer long read third generation sequencing platforms such as Single Molecule Real-Time (SMRT) DNA sequencing have partially resolved this, though the high error rates of long read data can also confound accurate assembly [6, 7]. The accuracy of a final assembly is determined by a combination of the complexity of the genome being sequenced, the quality of data used and the assembly approach [8].

For large, repetitive and complex genomes, whole genome shotgun (WGS) assembly usually results in highly fragmented assemblies that require considerable effort to order and orientate to produce acceptable pseudo molecules. In addition to the incorporation of MP and long read data, approaches may include the use of genetic and physical maps [9, 10], synteny to closely related species with reference genomes [4, 11], genotyping by sequencing [12] and population sequencing [13].

The more traditional BAC-by-BAC approaches to genome sequencing generally produce much higher quality assemblies than WGS, however BAC sequencing remains relatively expensive due to the cost of making BAC libraries, fingerprinting BAC clones and the sequencing of large numbers of overlapping BACs. Sequencing of the 2.5 Gbp maize genome using a BAC-by-BAC approach was estimated to cost US$50 million [14–16].

Bread wheat and sugarcane both have large and complex genomes which are challenging to assemble. Bread wheat has a hexaploid genome (2n = 6x = 42) that contains three ancestral diploid genomes (AABBDD), each with 7 chromosomes. The genome is large, around 17 Gbp and is predominantly made up of repeat elements [17, 18]. Sugarcane varieties have smaller genomes, around 10 Gbp [8, 19] but most are hybrids of two species, *Saccharum spontaneum* and *Saccharum officinarum*, *S. officinarum* being an octoploid with 2n = 80 chromosomes and *S. spontaneum* demonstrating varying ploidy (5–16x) and 2n chromosome number ranging from 40 to 128.

The complexity of the bread wheat and sugarcane genomes makes producing reference genome assemblies a challenge. While BAC-by-BAC approaches offer complexity reduction, individual BACs would still contain a high percentage of repeats (~80 %) in wheat as repeats are distributed in the genome. Thus BACs generated from such genomes are referred to as complex and repetitive despite the reduction in size. Decreasing the cost of BAC sequencing while maintaining or improving the accuracy of assembly has the potential to significantly decrease the cost of sequencing these genomes and enables the application of BAC-by-BAC approaches to diverse species. We present an efficient and cost effective approach for the sequencing and assembly of complex BACs using an optimised BAC pooling, data generation and bioinformatics assembly pipeline, and demonstrate the use of this approach by assembling BACs from bread wheat chromosome arm 7DS and sugarcane.

## Results and discussion

### Determination of the optimal sequencing depth for BAC assembly

**Fig. 1:**
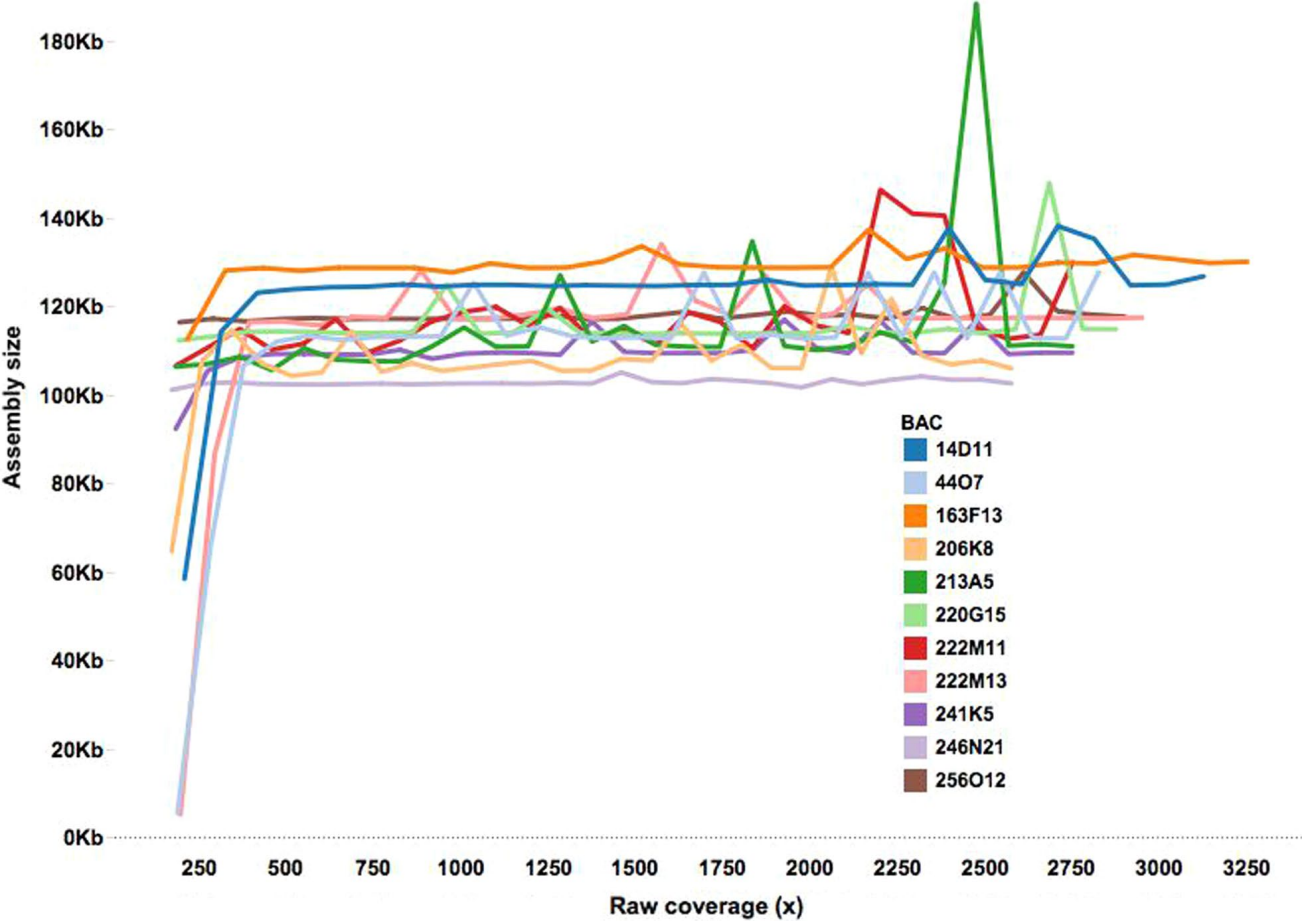
Optimal coverage for assembly. Assembly sizes vs coverage for each of the 11 sugarcane BACs. Assembly sizes peak at 450x and level off despite increase in coverage beyond 1500x

To determine the level of sequence coverage for accurate BAC assembly, eleven individual sugarcane BACs from the sugarcane cultivar R570 BAC library [20] were sequenced to extremely high coverage (>3000x). Reads were split into subsets representing 200x–3000x coverage, with 100x increments. The subsets were assembled with SASSY [21] (https://github.com/minillinim/SaSSY), which is an assembler customised for the assembly of complex repetitive BACs. Assemblies had an average N50 of 52 Kb and average number of contigs per BAC of 5.2 (Additional file 1: Table S1). For each of the BACs, assembly length increased until around 450x, then levelled off until 900x (Fig. 1). This suggests that >450x coverage is required for optimal BAC assembly, consistent with previous findings [21] in which the SASSY assembler was demonstrated to require a relatively large amount of data. The variation in assembly length observed for datasets greater than 900x (Fig. 1) is likely to be due to the increase in number of erroneous reads confounding the assembly process.

### Assessing the accuracy of BAC pooled assemblies

Even with the high degree of indexing available with Illumina DNA sequencing methods, the sequencing of individual BACs remains expensive. A pooling strategy was consequently established to increase throughput and reduce costs. The number of BACs which can be sequenced in a single lane of Illumina HiSeq 2000 is determined by the coverage required (450x–900x), the mean BAC length (around 120 Kb) and the data volume from the Illumina HiSeq (around 40 Gbp per lane). This suggests that pooling 384 BACs within a single lane, with accurate quantification and normalisation should produce around 850x coverage for each BAC. Considering that BAC DNA is likely to contain some contamination with *Escherichia coli* genomic sequence, the actual sequence coverage is likely to be less than this and fitting well within the range of 450x–900x shown to produce optimal assemblies.

To assess the accuracy of assembling bread wheat BACs in pools, single BACs were assembled and compared to the same BACs assembled as pools. Seven non-overlapping bread wheat BACs from chromosome 7DS were sequenced resulting in a sequence coverage range of 709x–1041x and a mean of 844x (Additional file 1: Table S2). After *E. coli* and vector sequences were filtered, sequence coverage ranged from 519x to 773x with a mean of 658x. Assemblies of the seven individual BACs (A, B, C, E, F, G and H) had an average N50 of 78 Kb with an average of four contigs per BAC (Table 1). Two BACs, B and G assembled as a single contig. Assemblies of pooled BACs (ABCE, BCEF, CEFG and EFGH) (Table 1) had an average N50 of 41 Kb with an average of 5.3 contigs per BAC.

**Table 1:** Assembly statistics of seven single bread wheat BACs and simulated BAC pool assemblies

BAC samples			Pre-processing statistics			Assembly statistics			
Name	Coverage x^a^	Vector %	*E.coli* %	Clonal %	Coverage x^b^	Contigs	N50 Kb	Longest Kb	Length Kb
A	811	4	13	0.6	643	4	99	99	113
B	1041	5	10	0.8	844	1	118	118	118
C	709	5	10	0.6	572	7	23	50	115
E	833	4	14	0.5	656	4	81	81	128
F	748	4	11	0.6	599	5	32	46	111
G	943	5	10	0.8	773	1	102	102	102
H	829	4	28	0.4	519	4	90	90	113
ABCE	849	4	12	0.6	679	23	43	97	452
BCEF	833	5	11	0.6	668	21	43	97	443
CEFG	808	4	11	0.6	650	22	32	81	433
EFGH	838	4	16	0.6	637	20	46	81	430

BACs A, B, C, E, F, G and H assembled individually and in simulated pools ABCE, BCEF, CEFG and EFGH

^a^Raw coverage estimated at 120 Kb prior to assembly

^b^Final coverage estimated at 120 Kb

**Fig. 2:**
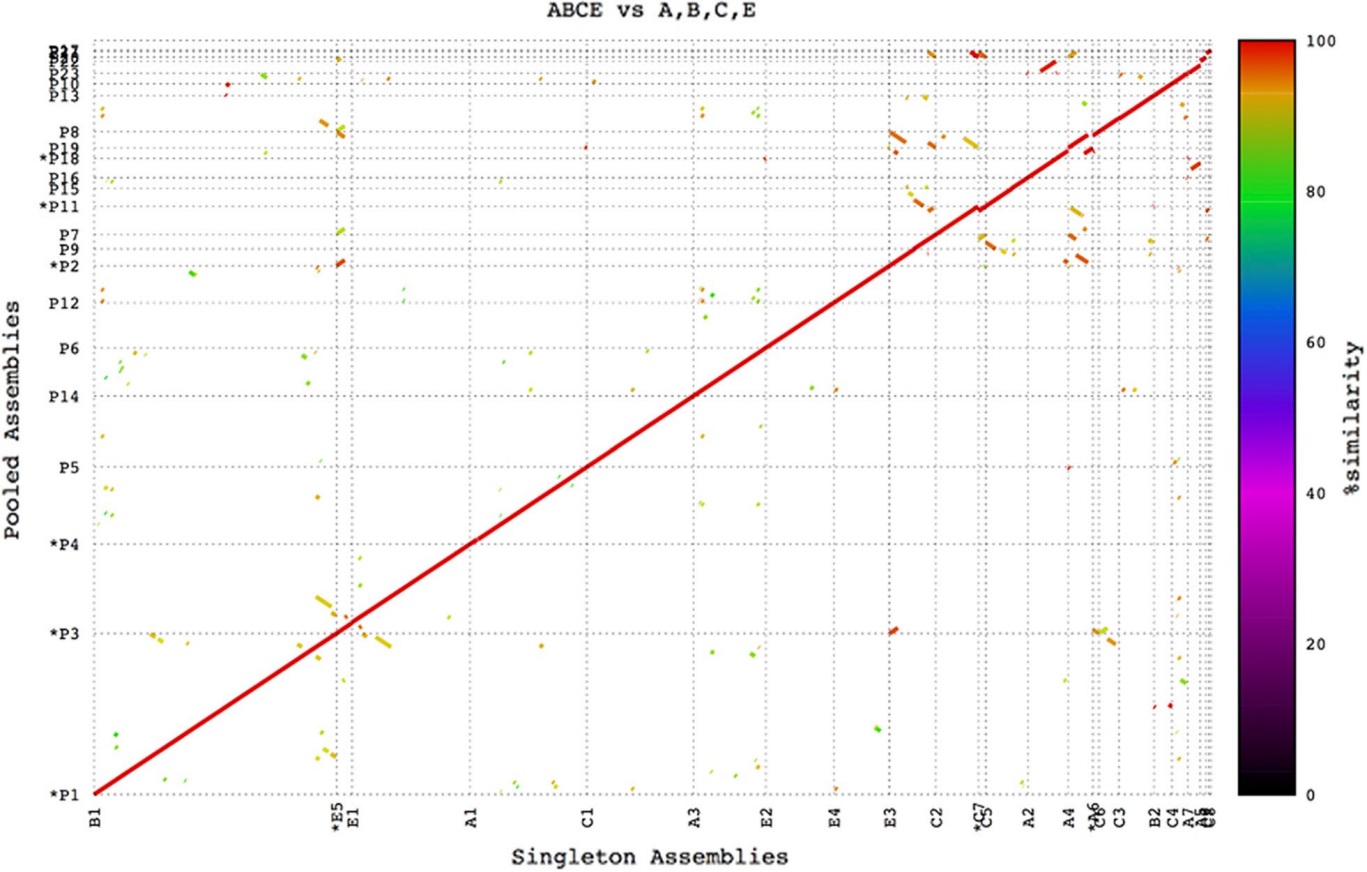
Mummer plot of assemblies of single BACs A, B, C, E against pooled BACs of ABCE

**Fig. 3:**
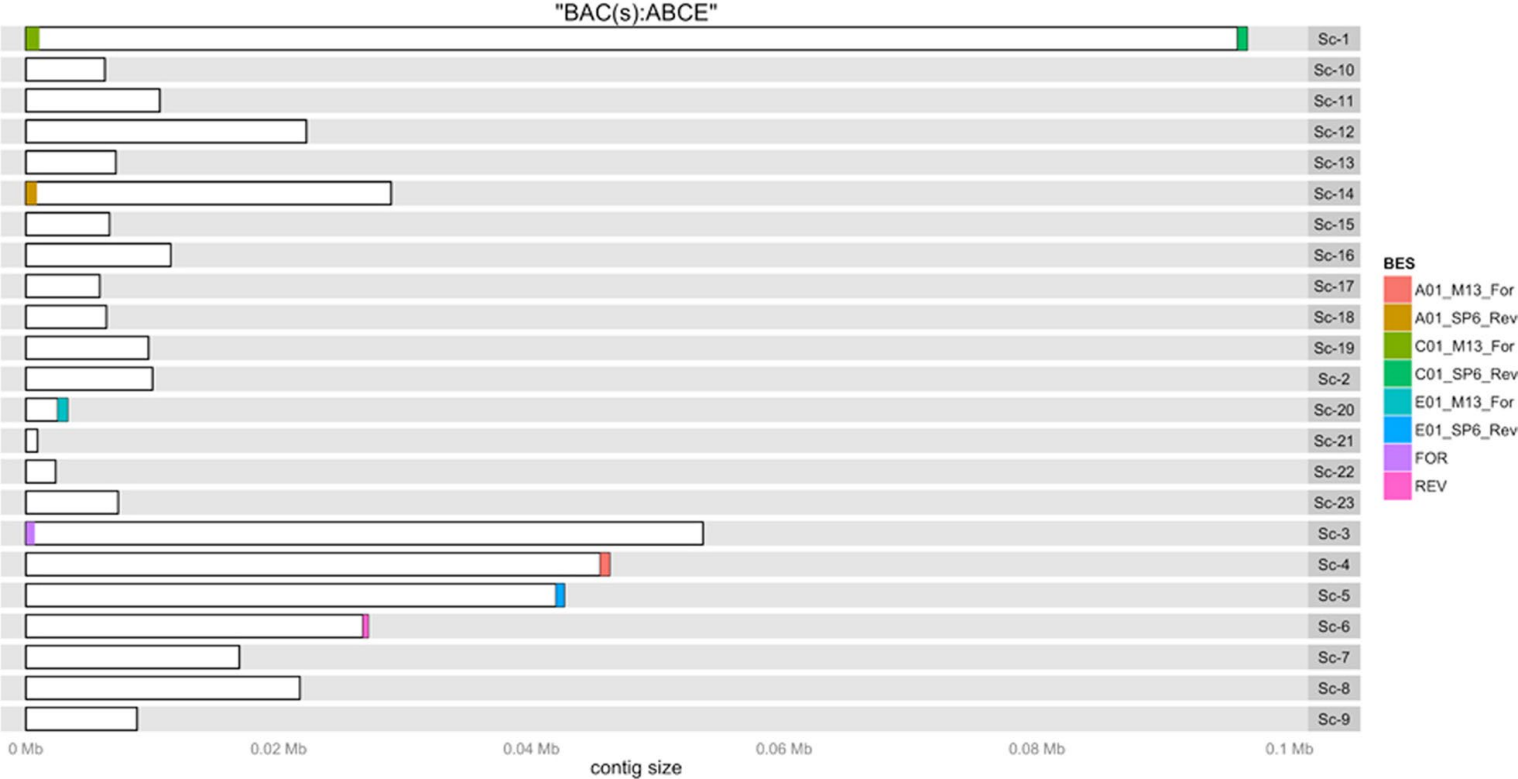
BES mappings on contigs of simulated pool (ABCE). Clones A, C and E have forward (M13_For) and reverse (SP6_Rev) BES (A01_M13_For, A01_SP6_Rev, C01_M13_For, C01_SP6_Rev, E01_M13_For, E01_SP6_Rev) respectively correctly mapped. Clone B had no BES available but 120 bp sequences from cloning vector ends (FOR and REV) were used to identify contig ends of clone B

A sequence comparison of contigs from individually assembled BACs (Additional file 1: Table S3) showed the integrity of individual BAC assemblies in pooled assemblies was maintained and assemblies of BAC pools remained collinear with those of individual BAC assemblies (Fig. 2). Pooled assemblies were further validated by comparison with their Sanger sequenced BAC ends. Mappings of BAC ends showed individual BACs remained separate in a pooled assembly (Fig. 3).

### High throughput wheat BAC assembly

**Fig. 4:**
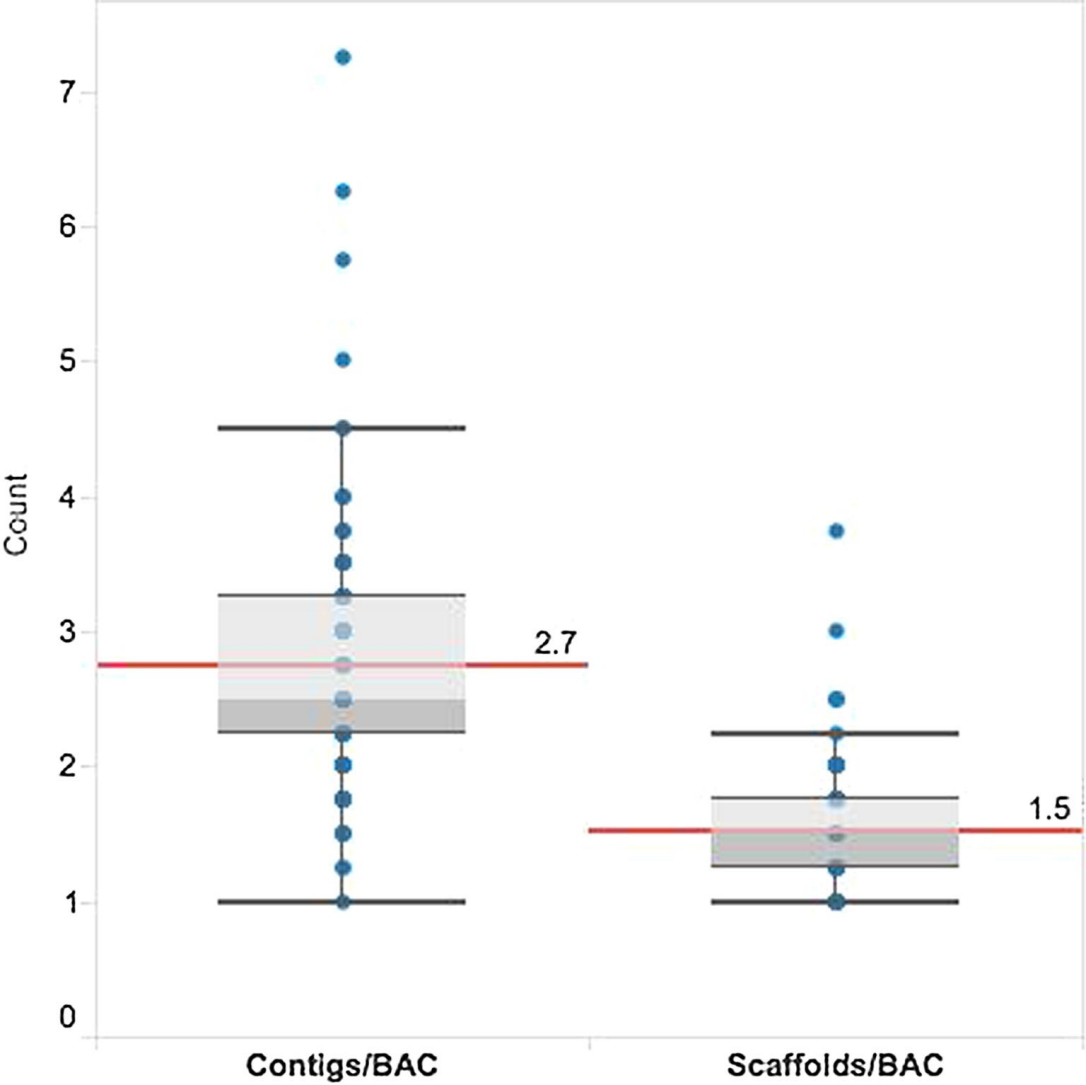
Distribution of no of contigs and scaffolds per BAC for 96 BAC pools

Following an assessment of the sequencing depth and pooling strategy, 96 BAC pools, each representing four randomly selected BACs from a bread wheat 7DS BAC library [22] were indexed and sequenced using a single lane of Illumina HiSeq 2000. *E coli* and vector sequences were removed resulting in a mean coverage per BAC of 690x with a range of between 184x and 889x. Only 3 % (12/384) of the BACs had coverage below 490x. Data from BAC pools was assembled using SASSY. The resulting assemblies (Table 2) had a mean N50 of 80 Kb, with an average of 2.7 contigs per BAC (Fig. 4). An average of 2.7 contigs per BAC for 96 pools compared to 5.3 contigs per BAC for four BAC pools (ABCE, BCEF, CEFG and EFGH) (Table 1) was lower and more accurate as a result of the higher number of BACs assembled. Of all the BACs, 99.5 % (382/384) had seven contigs or less per BAC, while 75 % of the BACs (288/384) had three contigs or less per BAC (Fig. 4; Additional file 1: Table S3). Assemblies were further improved by scaffolding with mate pair (MP) reads. Scaffolding resulted in an increase in N50 from 80 to 106 Kb. The average number of contigs per BAC after scaffolding was reduced from 2.7 to 1.5 (Fig. 4). After scaffolding, 99.5 % (382/384) of the BACs had four scaffolds or less per BAC (Fig. 4; Additional file 1: Table S4), while 75 % of the BACs (288/384) had two scaffolds or less per BAC (Fig. 4; Additional file 1: Table S4).

**Table 2:** Mate pair mapping orientations on *E. coli*, contigs and scaffolds

Orientation	Reference	% of pairs	Median insert size (Kb)
RF	*E coli*	99	6
	Contigs	99	6
	Scaffolds	97	6
FR	*E coli*	0.3	4
	Contigs	0.6	8
	Scaffolds	2	98
FF/RR	*E coli*	0.7	3
	Contigs	0.8	3
	Scaffolds	0.9	4

**Fig. 5:**
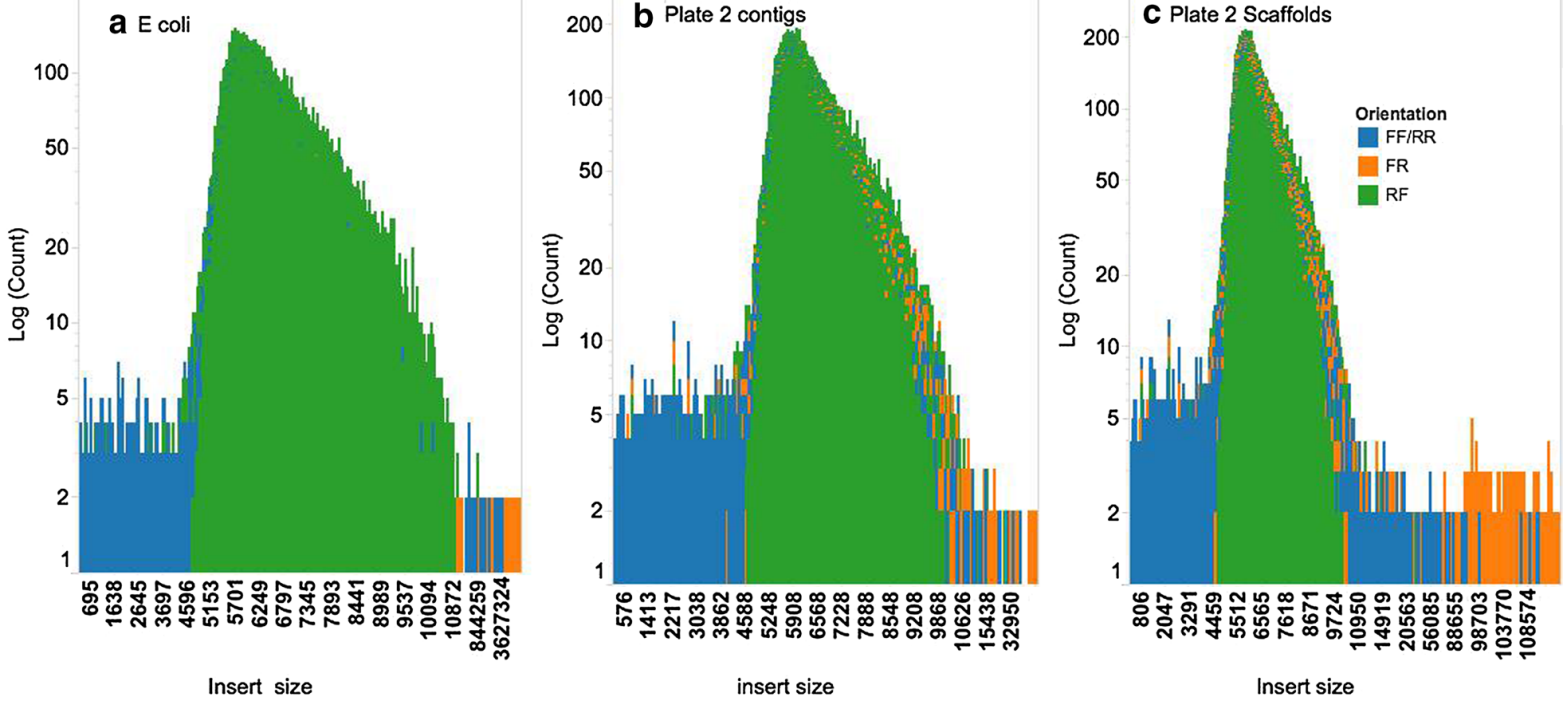
Distribution and orientation of MP insert sizes on *E coli* (**a**), contigs (**b**) and scaffolds (**c**) of 96 wheat BAC pools. *Y axis* (MP read counts in log scale), *X axis* (insert sizes). Correctly orientated MP reads with orientation RF (< –, – >) are shown in *green*, shadow library MP reads mapping with orientation FR (– >, < –) are shown in orange and chimeric MP reads mapping with orientation FF (– >, – >) and RR (< –, < –) are shown in *blue*

Paired read orientations and insert sizes of MP reads mapped to *E coli* and the 96 pool assemblies showed 99 % of the MP reads mapped with the expected MP orientation (RF) and expected insert size of 6 Kb (Fig. 5; Table 2). Scaffolds of the 96 pools had 97 % of the MP reads mapping with the expected MP orientation (RF) and expected insert size of 6 Kb. Shadow library and chimeric MP mapping orientations (FR), (FF and RR) respectively were altogether <3 % (Table 2) on both *E coli*, the 96 pool assemblies and scaffolds. This was within the expected values for Illumina Nextera MP libraries of ~2 % [23]. This suggests the contiguity of the assemblies is accurate.

A comparison of assembly sizes of the 96 pooled BACs to that of the sum of their corresponding individual BAC sizes estimated by fingerprinted contigs (FPC) software (Table 2) showed the average assembly size for a pool of four BACs was 441 Kb while the average predicted FPC size was 440 Kb. It is expected that assembly size and FPC size estimates would not be equal as repeats influence assembly sizes while FPC size estimates are approximations derived from the number of visualized restriction fragments. Despite this, paired t tests showed there was no significant difference between the FPC sizes and assembly sizes of the 96 pooled BACs (t = −0.14, df = 95, p > 0.8870).

While previous studies in barley recommended the use of read lengths >600 bp sequenced by Roche/454 [24], no current studies have demonstrated accurate robust pooled BAC assemblies using Illumina short reads in wheat. Our results show accurate assemblies of highly repetitive and complex genomes can be achieved using Illumina short reads with <3 % chimeric assemblies compared to previous estimates of 24–47 % using Roche/454 [24].

## Conclusions

BAC-by-BAC approaches are currently the most accurate assembly approaches available for highly repetitive and polyploid plant genomes. Compared to shotgun sequencing, a generally reported limitation of BAC-by-BAC approaches is the cost. However, this comparison ignores additional costs incurred during gene cloning projects due to incomplete and highly fragmented whole genome assemblies and the cost of anchoring and improving the assemblies. We have shown through indexing pooled BACs, costs can be substantially reduced while generating high quality assemblies. Physical coverage is critical for accurate assembly. We have shown pools with uneven physical coverage can easily be identified, re-sequenced and re-assembled at sufficient coverage.

We are currently applying our assembly approach to completely sequence and assemble wheat chromosome arm 7DS, with possible future applications in sequencing and assembly of the other wheat chromosome arms. The availability of a complete and accurate wheat reference sequence will greatly accelerate gene cloning, facilitate evolution and functional studies and speed up crop-breeding programs by providing a solid basis for marker development.

We recommend using the SASSY assembler with >450x coverage and pooling and indexing four BACs to attain highly accurate and robust assemblies for complex highly repetitive genomes at a reduced cost.

## Methods

### Plant material

Sugarcane BAC clones were selected from a BAC library of sugarcane (*Saccharumspp*) cultivar R570 [20], consisting of 103, 296 BAC clones with an average insert size of 130 Kb.

A wheat (*Triticum aestivum*, L.) 7DS—specific BAC library, constructed from a flow-sorted 7DS arm of cv. Chinese Spring [22] and having average insert size of 113 Kb, was used as a source of wheat BAC clones. The clones were previously fingerprinted using the SNaPshot-based high-information-content-fingerprinting (HICF) technology [25] and a physical contig map (https://urgi.versailles.inra.fr/gb2/gbrowse/wheat_phys_7DS_v1/) was constructed using FPC software [26]. A minimal tiling path (MTP) of 4608 BAC clones was selected to represent the 7DS arm.

### BAC DNA isolation and sequencing

The sugarcane DNA isolation and BAC library construction was done using the R570 cultivar as described in [27, 28]. Sequencing libraries of individual clones were prepared using TruSeq DNA HT kit (Illumina) and sequenced on the Illumina HiSeq 2000 platform with an insert size or 300 bp and read length of 100 bp. The sequencing depth was between 7000x and 50,000x per BAC.

Wheat BAC DNAs were isolated using NucleoSpin 96 Flash kit (Macherey–Nagel, Düren, Germany). A total of seven randomly selected MTP BAC clones were used to individually prepare sequencing libraries using TruSeq DNA HT kit (Illumina). The libraries were sequenced on Illumina HiSeq 2000 with an insert size of 300 and 150 bp paired end reads resulting in between 709x–1041x coverage per BAC. A total of 384 MTP BACs (one plate) from the same library were pooled into 96 pools of four non overlapping BACs, indexed in 96 well plates and sequenced on Illumina HiSeq 2000 with an insert size of 500 and 150 bp paired end reads resulting in >500x coverage per BAC. A mate pair library was prepared from the same MTP plate using Nextera Mate Pair Sample Preparation Kit (Illumina) and sequenced on the Illumina HiSeq 2000, 6–10 Kb insert size, 150 bp read length and ~100x coverage per BAC.

Paired BAC end sequences (BES) were generated for all MTP BAC clones by Sanger sequencing using four primers in total: T7 (For), SP6 (Rev), M13 Forward and M13 Reverse.

### Estimation of optimum coverage

Appropriate sequencing depth for assembly was determined by assembling different coverages of 11 deep sequenced sugarcane BAC clones which ranged from 4000x–10,000x coverage. Subsets of estimated 200x–3000x coverage in 100-fold increments were generated for each of the 11 clones by random selection of read pairs with replacement from the sequenced datasets. A total of 319 datasets were thus assembled with SASSY using default parameters at read lengths of 70 bp. Data points with fold coverage between 200x and 1000x were fitted with a local regression model (LOESS) [29]. LOESS is a robust non-parametric regression technique implemented in the R statistical package (R version 3.0.2). LOESS fits linear regressions over a subset of localized data points while limiting over-fitting. The resulting LOESS regression model was based on a smoothing parameter of 0.75, degrees of 2, and 91 observations.

### Determination of assembly integrity and accuracy

Contigs from assemblies of simulated BAC pools and single BACs were compared using BLASTN [30] with default parameters (gap opening penalty 5, gap extension 2, match 1, mismatch 2, evalue 10 and word size 11). BLAST mappings were used to pair a contig from a simulated pool assembly to its corresponding singleton assembly. MUMmer3 [31] was likewise used to compare single BAC and pooled BAC assemblies using default parameters. BAC end sequences (BES) were used to evaluate assembled contigs and to de convolute clones from the pools. Due to the high repeat content of wheat, several BES mapped to multiple positions within contigs of an assembled pool with exact matches. To evaluate the assembly accuracy of contigs using BES mapping positions, it was necessary to determine the correct position of a BES as below. For each BES (*B*) with a hit on a contig with bit score *b* and shortest distance to the edge of that contig *d*_*s*_ where length of *B* > 120 bp and length of *B* < *X,* the correct position of *B* was determined by the highest score *S* of all hits of *B*. *S* was obtained by subtracting the shortest distance *d*_*s*_ from each hits’ bit score *b*. BLAST bit scores were used as they incorporate both % identity and aligned length. The selected BES mapping positions per pool were visualized using R scripts implemented using the R software package ggbio [32].

### High throughput assembly pipeline

Using a custom pipeline, 96 pools of four bread wheat BACs each were filtered for *E. coli* str. K-12 and the cloning vector pIndigoBAC5 using SOAP2 [33] with parameters -m 400, -x 600, -M 0, -r 1, -v 0. If a read mapped singly to either *E coli* or pIndigoBAC, the whole pair was filtered out. Clonal reads were estimated using a custom clonal removal script and filtered out. Filtered datasets were then assembled using the SASSY assembler [21]. Mate pair libraries were adapter filtered using Cutadapt [34] to remove external Illumina adapters followed by removal of internal adapters using NxTrim [35]. Adapter filtered MP reads were mapped to all assembled pools. Using Perl scripts, mapped MP reads were selected for scaffolding each of the 96 pools individually. When a read pair (Read A and B) both mapped to the same pool with 100 % sequence identity, and full read length, the pairs’ mapping positions on contigs and pool were registered and used to generate an SSPACE [36] tab file for scaffolding that pool. The pools were then scaffolded with SSPACE with parameters K = 10, insert size of 4–9 Kb. Adapter filtered reads were re-mapped to scaffolded contigs and *E coli* using BLAST. Perl scripts were used to evaluate mapping orientations of MP read pairs that mapped only once to the same contig, with 100 % sequence identity and read lengths >100 bp.

## Additional file

10.1186/s13007-016-0107-9 Assembly statistics of sugarcane BAC clones. **Table S2**. Assembly statistics of 96 wheat BAC pools. **Table S3**. Contig sequence comparison between individual assemblies and pooled assemblies. **Table S4**. Number of contigs and scaffolds per BAC in percentiles of 384 BACs.
