# Supplementary material for: An efficient approach to BAC based assembly of complex genomes
Source: Plant Methods. 2016 Jan 20;12:2. doi: 10.1186/s13007-016-0107-9 (PMC4719536; doi:10.1186/s13007-016-0107-9)
Supplement: Supplementary file 1 — 10.1186/s13007-016-0107-9 Assembly statistics of sugarcane BAC clones. Table S2. Assembly statistics of 96 wheat BAC pools. Table S3. Contig sequence comparison between individual assemblies and pooled assemblies. Table S4. Number of contigs and scaffolds per BAC in percentiles of 384 BACs. [file 13007_2016_107_MOESM1_ESM.docx]

Table S 1: Assembly statistics of sugarcane BAC clones.

|  |  |  |  |  |
| --- | --- | --- | --- | --- |
|  | *m* | *sd* | *min* | *max* |
|  |  |  |  |  |
| Coverage x | 859.74 | 488.01 | 103.75 | 1972.13 |
| No. contigs | 5.68 | 2.93 | 1.00 | 24.00 |
| Contig N50 Kb | 52.46 | 31.48 | 1.98 | 128.85 |
| Longest contig Kb | 59.21 | 26.36 | 2.05 | 128.85 |
| Assembly length Kb | 114.99 | 14.14 | 5.30 | 188.43 |
|  |  |  |  |  |

Mean (*m*), standard deviation (*sd*) minimum (*min*) and maximum (*max*) values for sequence coverage, contig counts, contig assembly N50, longest contig lengths and assembly lengths of 319 datasets of increasing coverage from 11 deep sequenced sugarcane BAC clones.

**Table S 2: Assembly statistics of 96 wheat BAC pools**

|  | | | | | | | | | | |
| --- | --- | --- | --- | --- | --- | --- | --- | --- | --- | --- |
| **BAC Pool** | **% *E.coli*** | **% Vector** | **Cov** | **No contigs** | **Contig N50 Kb** | **Assembly size Kb** | **Scaffolds** | **Scaffold N50 Kb** | **Scaffold size Kb** | **FPC Kb** |
| 7DS-2-A1-C1-E1-G1-1 | 5.8 | 5 | 671.9 | 8 | 102 | 357 | 7 | 102 | 360 | 405 |
| 7DS-2-A2-C2-E2-G2-1 | 6.1 | 5.4 | 750.5 | 16 | 55 | 499 | 8 | 98 | 498 | 436 |
| 7DS-2-A3-C3-E3-G3-1 | 6.6 | 4.9 | 638.6 | 6 | 105 | 425 | 6 | 105 | 425 | 503 |
| 7DS-2-A4-C4-E4-G4-1 | 5 | 4.8 | 818 | 6 | 105 | 425 | 6 | 105 | 425 | 408 |
| 7DS-2-A5-C5-E5-G5-1 | 5.9 | 5.3 | 572.5 | 11 | 66 | 406 | 8 | 96 | 406 | 463 |
| 7DS-2-A6-C6-E6-G6-1 | 5.1 | 5.1 | 609.8 | 8 | 97 | 422 | 5 | 107 | 427 | 550 |
| 7DS-2-A7-C7-E7-G7-1 | 5.7 | 5.2 | 720.7 | 10 | 69 | 400 | 7 | 93 | 400 | 439 |
| 7DS-2-A8-C8-E8-G8-1 | 5.7 | 4.9 | 889.1 | 8 | 109 | 509 | 4 | 112 | 517 | 449 |
| 7DS-2-A9-C9-E9-G9-1 | 4.7 | 5 | 682.6 | 12 | 50 | 433 | 5 | 111 | 441 | 477 |
| 7DS-2-A10-C10-E10-G10-1 | 4.7 | 4.6 | 678.8 | 6 | 119 | 466 | 4 | 119 | 466 | 464 |
| 7DS-2-A11-C11-E11-G11-1 | 5.3 | 5.5 | 668.1 | 9 | 91 | 386 | 6 | 106 | 394 | 405 |
| 7DS-2-A12-C12-E12-G12-1 | 4.3 | 4.8 | 710.9 | 6 | 112 | 437 | 4 | 112 | 446 | 408 |
| 7DS-2-A13-C13-E13-G13-1 | 5.1 | 4.8 | 694.2 | 13 | 64 | 445 | 7 | 108 | 454 | 393 |
| 7DS-2-A14-C14-E14-G14-1 | 4 | 4.7 | 740.6 | 11 | 101 | 443 | 7 | 107 | 448 | 469 |
| 7DS-2-A15-C15-E15-G15-1 | 5.9 | 5.3 | 725.3 | 11 | 75 | 406 | 7 | 99 | 411 | 440 |
| 7DS-2-A16-C16-E16-G16-1 | 6.7 | 4.5 | 717.1 | 9 | 92 | 452 | 6 | 114 | 452 | 504 |
| 7DS-2-A17-C17-E17-G17-1 | 5.5 | 4.9 | 602.1 | 10 | 64 | 456 | 7 | 110 | 456 | 456 |
| 7DS-2-A18-C18-E18-G18-1 | 6.4 | 4.9 | 685.5 | 13 | 63 | 441 | 6 | 115 | 450 | 457 |
| 7DS-2-A19-C19-E19-G19-1 | 5.5 | 4.9 | 798.3 | 13 | 74 | 551 | 7 | 113 | 565 | 479 |
| 7DS-2-A20-C20-E20-G20-1 | 5 | 4.7 | 692.2 | 10 | 76 | 447 | 6 | 114 | 452 | 477 |
| 7DS-2-A21-C21-E21-G21-1 | 5.8 | 5 | 662.4 | 10 | 92 | 451 | 5 | 100 | 451 | 423 |
| 7DS-2-A22-C22-E22-G22-1 | 4.7 | 5.2 | 726.9 | 10 | 84 | 410 | 7 | 98 | 416 | 375 |
| 7DS-2-A23-C23-E23-G23-1 | 6 | 4.9 | 719.2 | 20 | 54 | 531 | 10 | 100 | 542 | 437 |
| 7DS-2-A24-C24-E24-G24-1 | 4.9 | 5 | 754.3 | 7 | 109 | 428 | 4 | 109 | 429 | 429 |
| 7DS-2-B1-D1-F1-H1-1 | 4.9 | 5.1 | 622.8 | 12 | 61 | 426 | 6 | 110 | 430 | 420 |
| 7DS-2-B2-D2-F2-H2-1 | 5.8 | 4.9 | 663.7 | 11 | 64 | 398 | 5 | 112 | 409 | 453 |
| 7DS-2-B3-D3-F3-H3-1 | 5.3 | 5 | 675.7 | 8 | 106 | 429 | 6 | 104 | 434 | 403 |
| 7DS-2-B4-D4-F4-H4-1 | 4.7 | 5.3 | 690.9 | 18 | 50 | 489 | 6 | 97 | 512 | 420 |
| 7DS-2-B5-D5-F5-H5-1 | 8.4 | 4.9 | 774.3 | 12 | 58 | 408 | 10 | 94 | 408 | 396 |
| 7DS-2-B6-D6-F6-H6-1 | 5.5 | 5 | 612.8 | 12 | 62 | 432 | 12 | 62 | 432 | 339 |
| 7DS-2-B7-D7-F7-H7-1 | 6.5 | 4.9 | 661.7 | 4 | 111 | 438 | 4 | 111 | 438 | 418 |
| 7DS-2-B8-D8-F8-H8-1 | 4.4 | 4.9 | 747.1 | 14 | 79 | 555 | 7 | 111 | 559 | 412 |
| 7DS-2-B9-D9-F9-H9-1 | 4.3 | 5.3 | 643.9 | 14 | 56 | 386 | 7 | 95 | 400 | 420 |
| 7DS-2-B10-D10-F10-H10-1 | 5.3 | 5.3 | 669.4 | 11 | 105 | 416 | 8 | 106 | 416 | 463 |
| 7DS-2-B11-D11-F11-H11-1 | 6.2 | 5 | 688.9 | 13 | 61 | 414 | 6 | 96 | 417 | 403 |
| 7DS-2-B12-D12-F12-H12-1 | 4.4 | 5 | 728 | 12 | 54 | 418 | 6 | 115 | 426 | 406 |
| 7DS-2-B13-D13-F13-H13-1 | 7 | 5 | 666.8 | 10 | 108 | 425 | 5 | 109 | 429 | 447 |
| 7DS-2-B14-D14-F14-H14-1 | 5.4 | 5.8 | 652.3 | 12 | 52 | 403 | 7 | 91 | 420 | 337 |
| 7DS-2-B15-D15-F15-H15-1 | 5.1 | 5.1 | 748.2 | 8 | 95 | 422 | 5 | 111 | 431 | 507 |
| 7DS-2-B16-D16-F16-H16-1 | 4.8 | 4.9 | 793.9 | 7 | 107 | 436 | 6 | 109 | 440 | 440 |
| 7DS-2-B17-D17-F17-H17-1 | 6.2 | 5.6 | 523.7 | 11 | 96 | 382 | 7 | 96 | 391 | 425 |
| 7DS-2-B18-D18-F18-H18-1 | 5.9 | 4.8 | 685.9 | 7 | 105 | 436 | 4 | 109 | 438 | 372 |
| 7DS-2-B19-D19-F19-H19-1 | 4.8 | 4.8 | 628.2 | 7 | 116 | 437 | 4 | 116 | 442 | 496 |
| 7DS-2-B20-D20-F20-H20-1 | 6.5 | 5.9 | 659.1 | 9 | 87 | 388 | 5 | 116 | 389 | 398 |
| 7DS-2-B21-D21-F21-H21-1 | 5.6 | 4.6 | 683.3 | 12 | 85 | 468 | 5 | 118 | 479 | 491 |
| 7DS-2-B22-D22-F22-H22-1 | 4.2 | 4.9 | 682.7 | 23 | 65 | 477 | 15 | 103 | 481 | 474 |
| 7DS-2-B23-D23-F23-H23-1 | 4.2 | 5 | 751.2 | 9 | 103 | 533 | 8 | 102 | 533 | 447 |
| 7DS-2-B24-D24-F24-H24-1 | 4.3 | 5.1 | 709.8 | 8 | 98 | 419 | 4 | 111 | 425 | 412 |
| 7DS-2-I1-K1-M1-O1-1 | 6.2 | 5.1 | 183.8 | 25 | 29 | 400 | 5 | 110 | 413 | 483 |
| 7DS-2-I2-K2-M2-O2-1 | 4.9 | 4.9 | 836.6 | 5 | 109 | 411 | 4 | 108 | 415 | 459 |
| 7DS-2-I3-K3-M3-O3-1 | 6 | 5 | 677 | 15 | 51 | 525 | 5 | 108 | 530 | 560 |
| 7DS-2-I4-K4-M4-O4-1 | 4.9 | 5.2 | 730.2 | 11 | 65 | 521 | 6 | 112 | 527 | 386 |
| 7DS-2-I5-K5-M5-O5-1 | 4.7 | 5.3 | 641.5 | 9 | 94 | 422 | 5 | 112 | 427 | 430 |
| 7DS-2-I6-K6-M6-O6-1 | 5.1 | 5.8 | 750.1 | 9 | 81 | 492 | 5 | 103 | 502 | 405 |
| 7DS-2-I7-K7-M7-O7-1 | 4.8 | 4.8 | 746.4 | 7 | 100 | 439 | 6 | 115 | 443 | 516 |
| 7DS-2-I8-K8-M8-O8-1 | 5.1 | 4.9 | 815.4 | 16 | 56 | 455 | 7 | 120 | 460 | 471 |
| 7DS-2-I9-K9-M9-O9-1 | 4.8 | 5.1 | 201.3 | 29 | 19 | 397 | 4 | 103 | 409 | 626 |
| 7DS-2-I10-K10-M10-O10-1 | 4.4 | 5.5 | 755.2 | 5 | 102 | 395 | 4 | 104 | 400 | 410 |
| 7DS-2-I11-K11-M11-O11-1 | 4.7 | 4.8 | 673.2 | 16 | 80 | 525 | 4 | 113 | 531 | 528 |
| 7DS-2-I12-K12-M12-O12-1 | 3.9 | 5.1 | 680 | 9 | 88 | 428 | 5 | 109 | 435 | 406 |
| 7DS-2-I13-K13-M13-O13-1 | 3.9 | 5 | 771.4 | 9 | 106 | 457 | 7 | 116 | 462 | 403 |
| 7DS-2-I14-K14-M14-O14-1 | 4.6 | 4.9 | 726.9 | 12 | 62 | 458 | 6 | 115 | 462 | 473 |
| 7DS-2-I15-K15-M15-O15-1 | 4.7 | 5.2 | 705.9 | 10 | 70 | 428 | 5 | 108 | 433 | 452 |
| 7DS-2-I16-K16-M16-O16-1 | 5.2 | 5 | 677.9 | 15 | 68 | 399 | 7 | 102 | 402 | 386 |
| 7DS-2-I17-K17-M17-O17-1 | 5.1 | 5.8 | 698.8 | 9 | 105 | 393 | 4 | 110 | 397 | 393 |
| 7DS-2-I18-K18-M18-O18-1 | 5.2 | 5 | 705 | 12 | 51 | 439 | 6 | 104 | 441 | 422 |
| 7DS-2-I19-K19-M19-O19-1 | 5.6 | 5 | 672.9 | 7 | 87 | 447 | 5 | 119 | 452 | 429 |
| 7DS-2-I20-K20-M20-O20-1 | 5.2 | 5 | 724.9 | 13 | 75 | 429 | 8 | 105 | 430 | 443 |
| 7DS-2-I21-K21-M21-O21-1 | 5.9 | 4.8 | 729.6 | 10 | 84 | 457 | 6 | 121 | 470 | 378 |
| 7DS-2-I22-K22-M22-O22-1 | 5.4 | 4.9 | 678.3 | 14 | 83 | 433 | 8 | 105 | 446 | 430 |
| 7DS-2-I23-K23-M23-O23-1 | 5.3 | 4.7 | 700.5 | 9 | 118 | 432 | 4 | 118 | 440 | 381 |
| 7DS-2-I24-K24-M24-O24-1 | 5.6 | 4.4 | 731.5 | 13 | 98 | 557 | 5 | 119 | 559 | 605 |
| 7DS-2-J1-L1-N1-P1-1 | 7.3 | 4.9 | 716.7 | 11 | 100 | 431 | 5 | 107 | 436 | 405 |
| 7DS-2-J2-L2-N2-P2-1 | 3.9 | 4.8 | 760 | 9 | 59 | 451 | 5 | 103 | 450 | 463 |
| 7DS-2-J3-L3-N3-P3-1 | 5.2 | 4.9 | 742.8 | 9 | 85 | 432 | 5 | 117 | 441 | 456 |
| 7DS-2-J4-L4-N4-P4-1 | 5.6 | 5.2 | 598.7 | 14 | 56 | 426 | 5 | 113 | 444 | 364 |
| 7DS-2-J5-L5-N5-P5-1 | 4.7 | 4.7 | 806.5 | 10 | 83 | 468 | 6 | 111 | 473 | 409 |
| 7DS-2-J6-L6-N6-P6-1 | 4.5 | 5 | 739.6 | 7 | 108 | 420 | 6 | 108 | 424 | 467 |
| 7DS-2-J7-L7-N7-P7-1 | 4.7 | 5 | 735.5 | 10 | 64 | 437 | 5 | 112 | 450 | 429 |
| 7DS-2-J8-L8-N8-P8-1 | 3.8 | 5.1 | 672.2 | 11 | 70 | 433 | 7 | 103 | 437 | 412 |
| 7DS-2-J9-L9-N9-P9-1 | 5.3 | 4.8 | 709.6 | 11 | 103 | 427 | 6 | 113 | 436 | 381 |
| 7DS-2-J10-L10-N10-P10-1 | 4 | 5.2 | 735.4 | 14 | 46 | 435 | 6 | 111 | 444 | 402 |
| 7DS-2-J11-L11-N11-P11-1 | 5.3 | 5.1 | 769.4 | 15 | 64 | 535 | 7 | 104 | 539 | 466 |
| 7DS-2-J12-L12-N12-P12-1 | 4.9 | 5.2 | 679 | 10 | 74 | 443 | 5 | 114 | 452 | 415 |
| 7DS-2-J13-L13-N13-P13-1 | 4.4 | 5.3 | 740.5 | 13 | 93 | 357 | 8 | 94 | 365 | 463 |
| 7DS-2-J14-L14-N14-P14-1 | 4.3 | 4.9 | 819.4 | 7 | 80 | 448 | 5 | 110 | 452 | 474 |
| 7DS-2-J15-L15-N15-P15-1 | 4.2 | 5.1 | 708.8 | 11 | 60 | 423 | 4 | 105 | 429 | 447 |
| 7DS-2-J16-L16-N16-P16-1 | 4 | 5.4 | 708.4 | 13 | 80 | 425 | 7 | 107 | 430 | 410 |
| 7DS-2-J17-L17-N17-P17-1 | 4.1 | 5.2 | 412.8 | 11 | 68 | 424 | 9 | 72 | 428 | 471 |
| 7DS-2-J18-L18-N18-P18-1 | 4.4 | 5 | 588.7 | 9 | 67 | 445 | 4 | 117 | 449 | 382 |
| 7DS-2-J19-L19-N19-P19-1 | 3.9 | 5.3 | 658.3 | 9 | 101 | 403 | 5 | 101 | 403 | 479 |
| 7DS-2-J20-L20-N20-P20-1 | 5.3 | 5 | 706 | 12 | 65 | 461 | 7 | 106 | 461 | 476 |
| 7DS-2-J21-L21-N21-P21-1 | 4.4 | 4.8 | 743.3 | 18 | 64 | 479 | 10 | 126 | 503 | 419 |
| 7DS-2-J22-L22-N22-P22-1 | 4 | 5.1 | 718.3 | 9 | 84 | 415 | 8 | 84 | 415 | 440 |
| 7DS-2-J23-L23-N23-P23-1 | 6.6 | 4.9 | 677.9 | 8 | 100 | 431 | 7 | 106 | 436 | 410 |
| 7DS-2-J24-L24-N24-P24-1 | 4.4 | 4.8 | 669.7 | 8 | 73 | 479 | 5 | 118 | 483 | 494 |
| Assembly statistics of 96 pools showing % or *E. coli* and vector sequences prior to filtering, final sequence coverage (cov), No. of contigs, contig N50 and each pools’ total assembly size. The number of contigs (scaffolds) after MP scaffolding with the corresponding scaffold N50, total scaffolded pool size (scaffold size) and each pools’ FPC size estimates are also shown. | | | | | | | | | | |

Table S 3: Contig sequence comparison between individual assemblies and pooled assemblies.

|  |  | **1** |  |  |  |  |  | **2** |  |  |  |  |  | **3** |  |  |  |  |  | **4** |  |  |
| --- | --- | --- | --- | --- | --- | --- | --- | --- | --- | --- | --- | --- | --- | --- | --- | --- | --- | --- | --- | --- | --- | --- |
|  |  | A/B/C/E |  |  |  |  |  | B/C/E/F |  |  |  |  |  | C/E/F/G |  |  |  |  |  | E/F/G/H |  |  |
|  |  |  |  |  |  |  |  |  |  |  |  |  |  |  |  |  |  |  |  |  |  |  |
| ***a*** | ***b*** | % homology | Length | ***m*** |  | ***a*** | ***b*** | % homology | Length | ***m*** |  | ***a*** | ***b*** | % homology | Length | ***m*** |  | ***a*** | ***b*** | % homology | Length | ***m*** |
| A1 | 4 | 99.96 | 43248 | 18 |  | B1 | 2 | 99.99 | 96619 | 5 |  | C1 | 4 | 100 | 42588 | 0 |  | E1 | 4 | 100 | 46900 | 0 |
| A2 | 16 | 99.57 | 11460 | 46 |  | C1 | 3 | 100 | 42588 | 0 |  | C2 | 7 | 99.28 | 16897 | 79 |  | E2 | 7 | 100 | 27071 | 0 |
| A3 | 14 | 100 | 28897 | 0 |  | C2 | 4 | 99.28 | 16897 | 79 |  | C3 | 5 | 99.84 | 14034 | 22 |  | E3 | 5 | 99.85 | 18448 | 28 |
| A4 | 19 | 99.95 | 7900 | 4 |  | C3 | 5 | 100 | 14034 | 0 |  | C4 | 1 | 100 | 6253 | 0 |  | E4 | 12 | 99.99 | 22192 | 2 |
| A5 | 22 | 100 | 2360 | 0 |  | C4 | 10 | 100 | 6253 | 0 |  | C5 | 5 | 99.91 | 10616 | 10 |  | F1 | 6 | 99.99 | 45618 | 5 |
| A7 | 23 | 99.86 | 4368 | 6 |  | C5 | 11 | 99.91 | 10579 | 10 |  | C6 | 17 | 100 | 7972 | 0 |  | F2 | 8 | 100 | 31496 | 0 |
| B1 | 1 | 99.99 | 96619 | 5 |  | C6 | 5 | 100 | 7972 | 0 |  | C7 | 7 | 100 | 2967 | 0 |  | F3 | 11 | 99.99 | 11189 | 1 |
| C1 | 5 | 100 | 42588 | 0 |  | C7 | 4 | 100 | 2967 | 0 |  | E1 | 3 | 100 | 46900 | 0 |  | F4 | 15 | 100 | 5780 | 0 |
| C2 | 7 | 99.28 | 16897 | 79 |  | E1 | 1 | 100 | 46900 | 0 |  | E2 | 8 | 100 | 27071 | 0 |  | F5 | 17 | 99.86 | 7160 | 5 |
| C3 | 8 | 100 | 14034 | 0 |  | E2 | 9 | 100 | 27071 | 0 |  | E3 | 9 | 100 | 10014 | 0 |  | G1 | 3 | 100 | 81338 | 0 |
| C4 | 10 | 100 | 6253 | 0 |  | E3 | 7 | 100 | 10014 | 0 |  | E4 | 14 | 99.99 | 22192 | 0 |  | H1 | 9 | 100 | 69479 | 0 |
| C5 | 11 | 99.91 | 10579 | 10 |  | E4 | 14 | 100 | 22192 | 0 |  | F1 | 6 | 99.99 | 45618 | 0 |  | H2 | 13 | 100 | 9791 | 0 |
| C6 | 8 | 100 | 7972 | 0 |  | E1 | 8 | 100 | 6298 | 0 |  | F2 | 11 | 100 | 31496 | 2 |  | H3 | 14 | 100 | 7594 | 0 |
| C7 | 7 | 100 | 2967 | 0 |  | F1 | 6 | 99.97 | 45618 | 0 |  | F3 | 12 | 99.99 | 11189 | 0 |  | H4 | 1 | 99.81 | 13475 | 23 |
| C8 | 21 | 100 | 918 | 0 |  | F2 | 12 | 100 | 31496 | 12 |  | F4 | 16 | 100 | 5780 | 5 |  |  |  |  |  |  |
| C9 | 20 | 100 | 1114 | 0 |  | F3 | 13 | 100 | 11191 | 0 |  | F5 | 18 | 99.86 | 7160 | 0 |  |  |  |  |  |  |
| E1 | 3 | 100 | 46900 | 0 |  | F4 | 17 | 100 | 5780 | 0 |  | G1 | 2 | 100 | 81338 | 0 |  |  |  |  |  |  |
| E2 | 6 | 100 | 27071 | 0 |  | F5 | 6 | 99.99 | 7160 | 0 |  |  |  |  |  |  |  |  |  |  |  |  |
| E3 | 2 | 99.86 | 10014 | 14 |  |  |  |  |  |  |  |  |  |  |  |  |  |  |  |  |  |  |
| E4 | 12 | 100 | 22192 | 0 |  |  |  |  |  |  |  |  |  |  |  |  |  |  |  |  |  |  |
| Contigs from individual assemblies A, B, C, E, F, G and H (***a***) and contigs from pools of ABCE, BCEF, CEFG and EFGH (***b)*** were compared for % homology and base pair miss-matches *(****m****)****.*** | | | | | | | | | | | | | | | | | | | | | | |

Table S 4: Number of contigs and scaffolds per BAC in percentiles of 384 BACs.

| Percentile | Contigs per BAC | Scaffolds per BAC |
| --- | --- | --- |
| 99.5% | 7 | 4 |
| 97.5% | 6 | 3 |
| 90.0% | 4 | 2 |
| 75.0% | 3 | 2 |
| 50.0% | 3 | 2 |
| 25.0% | 2 | 1 |
| 10.0% | 2 | 1 |
| 2.5% | 1 | 1 |
| 0.5% | 1 | 1 |
